# Clinical features and radiographic findings in cats with eosinophilic, neutrophilic, and mixed airway inflammation (2011‐2018)

**DOI:** 10.1111/jvim.15772

**Published:** 2020-04-27

**Authors:** Elizabeth A. Lee, Lynelle R. Johnson, Eric G. Johnson, William Vernau

**Affiliations:** ^1^ William R. Pritchard Veterinary Medical Teaching Hospital University of California School of Veterinary Medicine Davis California USA; ^2^ Department of Medicine and Epidemiology University of California School of Veterinary Medicine Davis California USA; ^3^ Department of Surgical and Radiological Sciences University of California School of Veterinary Medicine Davis California USA; ^4^ Department of Pathology, Microbiology and Immunology University of California School of Veterinary Medicine Davis California USA

**Keywords:** asthma, endoscopy, eosinophilic inflammation, neutrophilic inflammation, parenchymal disease, respiratory tract

## Abstract

**Background:**

Idiopathic inflammatory airway disease (IAD) in cats often is described as asthmatic (eosinophilic) or bronchitic (neutrophilic), but this designation requires collection of airway fluid and it fails to consider cats with mixed airway inflammation.

**Objective:**

To identify clinical features that would differentiate inflammatory disease types.

**Animals:**

Forty‐nine cats with nonspecific airway inflammation identified by bronchoscopic bronchoalveolar lavage (BAL) between 2011 and 2018 were evaluated.

**Methods:**

This is a retrospective study. Cats were categorized by BAL differential cytology as having eosinophilic (eosinophils >20% with neutrophils <14%, or eosinophils >50%), mixed (eosinophils 20%‐50% and neutrophils >14% or discordant inflammation from 2 BAL sites), or neutrophilic (neutrophils >14% and eosinophils <20%) inflammation. Type and duration of presenting complaints, signalment, body condition score, respiratory rate, CBC results, bronchoscopy, BAL results (% recovery, total nucleated cell count, differential cell count), and radiographic findings were compared among groups.

**Results:**

Idiopathic IAD was diagnosed in 49 cats, with BAL eosinophilic inflammation in 23, mixed inflammation in 14, and neutrophilic inflammation in 12. Cough was the predominant presenting complaint with no difference in duration of signs among groups (median, 5.5 months). Respiratory rate and effort also did not differ. Cats with eosinophilic inflammation were significantly younger (4.4 ± 3.3 years) than those with neutrophilic (8.0 ±5.6 years) or mixed inflammation (7.5 ± 4.0 years; *P* = .03). Results of CBC and interpretation of radiographic findings did not differ among groups.

**Conclusions and Clinical Importance:**

Substantial overlap exists in clinical and radiographic findings in cats with various forms of idiopathic airway inflammation.

**Abbreviations**
BAL: bronchoalveolar lavage
BCS: body condition score
IAD: inflammatory airway disease
TNCC: total nucleated cell count

## 1 INTRODUCTION

Inflammatory airway disease (IAD) is common in cats and is an important differential diagnosis for cats presenting with a history of cough, wheezing, or increased expiratory effort.^1^, ^2^, ^3^, ^4^, ^5^ Inflammatory airway disease has been proposed to encompass 2 distinct syndromes: asthma and chronic bronchitis.^4^, ^6^, ^7^ Asthma in cats is considered to be similar to asthma in humans and is characterized by increased airway responsiveness and bronchoconstriction, manifested by acute wheezing or respiratory difficulty, which can be reversed by treatment with a bronchodilator. Similar to some forms of the syndrome in humans, asthma in cats has been suggested to result from exposure to allergens, and a disease syndrome typified by eosinophilic airway inflammation can be created experimentally by allergen or antigen challenge.^2^, ^3^, ^4^, ^7^ In contrast, chronic bronchitis in cats is defined by the presence of cough, and bronchoconstriction is not part of the clinical picture.^2^, ^8^ Chronic bronchitis in cats has been defined similarly to the disease in dogs^9^ with development of nonseptic suppurative airway inflammation in response to an unknown insult. Currently, the etiopathogenesis of the naturally occurring diseases remains poorly understood, and it is unclear whether asthma and chronic bronchitis in cats are separate diseases or part of a spectrum of diseases resulting from inflammation.

In people, differentiation of asthma from bronchitis relies on pulmonary function testing,^10^ which is not readily applicable to cats. Diagnosis of IAD is based on exclusion of other etiologies such as parasitic, infectious, or neoplastic disease in conjunction with a nonseptic inflammatory infiltrate on airway lavage sampling. Asthma in cats is considered to result in predominantly eosinophilic inflammation and chronic bronchitis in predominantly nonseptic neutrophilic inflammation. 4, 6, 7 However, marked airway eosinophilia has been reported in clinically normal cats,^11^, ^12^ and techniques used to collect and evaluate airway samples have been inconsistent across studies.^1^, ^2^, ^3^, ^4^, ^5^, ^7^, ^9^, ^12^, ^13^, ^14^

Previous studies evaluating clinical findings in cats with naturally occurring disease have failed to identify clinically relevant differences between cats with asthma and those with chronic bronchitis, with the exception of some investigators noting younger age in cats with airway eosinophilia.^1^, ^5^, ^8^, ^13^, ^15^ The purpose of our retrospective study was to investigate clinical variables that could aid in differentiation between inflammatory airway syndromes in a large group of cats with naturally occurring disease in which standardized diagnostic testing, including bronchoscopic bronchoalveolar lavage (BAL), had been performed. We hypothesized that cats with eosinophilic inflammation would be younger, have a shorter duration of clinical signs, display less severe radiographic changes, and have higher circulating eosinophil counts compared to those with neutrophilic or mixed airway inflammation.

## 2 MATERIALS AND METHODS

Medical records of cats undergoing bronchoscopy with BAL for evaluation of lower respiratory signs at the William R. Pritchard Veterinary Medical Teaching Hospital of the University of California, Davis between 1 January 2011 and 1 January 2019, were reviewed for evidence of idiopathic IAD. Inclusion criteria included lack of response to antimicrobials and a positive clinical response to corticosteroid treatment. Exclusion criteria included a final diagnosis of neoplasia, infection (bacterial, mycoplasmal, or fungal), parasitic bronchitis, foreign body, or trauma. In addition, cats that had BAL samples containing <300 cells/μL were excluded from analysis because of concerns about inadequate recovery of fluid that had been in contact with the epithelial lining. All medical records were reviewed by 2 authors (E.A.L. and L.R.J.).

Cats were premedicated using a balanced anesthetic plan determined by board‐certified anesthesiologists, and bronchoscopy was performed under IV propofol anesthesia by using an induction dosage of 4 mg/kg followed by 0.1 to 0.4 mg/kg/min as a continuous rate infusion. All cats were preoxygenated and treated with SC terbutaline (0.01 mg/kg) before the procedure, and oxygenation was maintained by jet ventilation at 180 breaths/min. Pulse oximetry, ECG, and blood pressure were monitored throughout the procedure in all cats. Bronchoscopy was performed using either a 2.8 mm × 70 cm videoendoscope with a 1.2‐mm channel (Karl Storz Flex XC, Goleta, California) or a 3.8 mm × 55 cm videoendoscope with a 1.2‐mm channel (Olympus BF3C160, Center Valley, Pennsylvania).

Bronchoscopy was performed in standard fashion by faculty and house officers trained by 1 of the authors (L.R.J.). All bronchi were evaluated sequentially, and then the bronchoscope was removed from the airways to irrigate the channel with sterile saline and wipe debris from the outer surface. The bronchoscope was reinserted to complete BAL at specific site(s) chosen by the endoscopist. Bronchoalveolar lavage was performed by instilling 3 to 5 mL of warmed, sterile saline through the biopsy channel of the endoscope, flushing the channel with 2 to 3 mL of air, and immediately applying hand suction to recover fluid that had been in contact with the bronchoalveolar space. Single‐aliquot lavage was performed at 1 to 3 sites and fluid was submitted for analysis within 1 hour of collection.

Bronchoalveolar lavage fluid from distinct lung sites was analyzed separately for determination of total and differential cell counts based on a count of 200 cells. A pooled BAL sample was submitted for aerobic and *Mycoplasma* cultures; anaerobic cultures were submitted at the discretion of the attending clinician. Total nucleated cell counts (TNCCs) were performed on unfiltered BAL using an automated cell counter (Advia 120, Siemens, Deerfield, Illinois) and reported as TNCC/μL. Slides were prepared for cytologic assessment by cytocentrifugation (Cytospin3, ThermoShandon, Pittsburgh, Pennsylvania) followed by Wright‐Giemsa staining using an automated cell stainer (Model 7151 Wescor Aerospray Hematology Pro, ELITech Bio‐Medical Systems, Logan, Utah).

Reference intervals used by our laboratory for BAL total and differential cell counts are 300 to 400 cells/μL comprised of up to 7% neutrophils or lymphocytes, up to 18% eosinophils, and 65% to 85% macrophages.^11^, ^12^ Cats were categorized as having eosinophilic, mixed, or neutrophilic inflammation based on BAL fluid differential cytology. Previous reports have used eosinophil percentages of >17 to 20^8^, ^15^, ^16^, ^17^, ^18^ to define eosinophilic inflammation, and neutrophil percentage exceeding the reference range to describe noneosinophilic or neutrophilic inflammation. Other studies define inflammation based on the prominent cell type. 1, 3, 19, 20 Rigorous categorizations were used here, and eosinophilic inflammation was defined as BAL fluid cytology containing eosinophils >20% with neutrophils <14% (mean + twice the SD to ensure that cytological inflammation was consistent with clinical disease^11^), or by eosinophils >50% in 1 to 2 BAL sites. Neutrophilic inflammation was defined by neutrophils >14% and eosinophils <20%. Mixed inflammation was defined by eosinophils 20% to 50% and neutrophils >14% on cytology or as discordant inflammation from 2 BAL sites.^16^ When only a single site was lavaged, cases were categorized based on interpretation of a single lavage fluid cytology.

Data collected from the medical record included type and duration of presenting clinical signs (e.g., cough, sneeze, nasal discharge, increased respiratory effort) as reported by the owner, age, sex, indoor or outdoor status, medications used within the last 7 days, weight, body condition score (BCS), physical examination findings (temperature, heart rate, respiratory rate, and effort), bronchoscopy findings, BAL fluid recovery, BAL fluid cell count and cytology, and BAL culture. Fecal and heartworm test results were recorded where available and contrasted with results from the hospital population. Radiographic features and CBC data were included only when performed at the University of California Davis School of Veterinary Medicine for consistency of technique, interpretation, and reference ranges. The CBC data were evaluated for total leukocyte, neutrophil, and eosinophil counts. Thoracic radiographs were reviewed in masked fashion by a board‐certified radiologist (E.G.J.) using a 0 to 9 scoring system adapted from previously described studies.^1^, ^4^, ^15^ Bronchial patterns were scored as follows: 0, absence of a bronchial pattern; 1, mild, first‐generation bronchi visible; 2, moderate, second‐generation bronchi visible; and 3, severe, third‐generation bronchi visible. Interstitial patterns were scored as follows: 0, absence of interstitial markings; 1, mild interstitial framework visible; 2, moderate, interstitial framework distinguishable from bronchiolar pattern; and 3, severe, overt interstitial framework. Alveolar patters were scored as follows: 0, absence of alveolar infiltrates; 1, mild, focal unilateral alveolar pattern; 2, moderate, focal bilateral alveolar pattern; and 3, severe, multifocal bilateral alveolar pattern or lobar collapse. Total possible severity score was 9. Additional features recorded included topographic distribution of disease, presence of bronchiectasis or broncholithiasis, pulmonary artery enlargement, and hyperinflation or hypoinflation.

Data were assessed for normality using the D'Agostino and Pearson Omnibus test (GraphPad Prism version 5.0f, San Diego, California). Parametric data are reported as mean with SD and nonparametric data as median with range. Normally distributed data (age, heart rate, radiographic score, neutrophil count, BAL fluid recovery) were compared among groups using a 1‐way analysis of variance with Tukey's multiple comparison test for post hoc analysis. Nonparametric data (weight, duration of signs, temperature, respiratory rate, white blood cell count, eosinophil count, BAL fluid TNCC/μL) were assessed using a Kruskal‐Wallis test and post hoc Dunns multiple comparison test. Presence of cough, sneeze, or nasal discharge, increased respiratory effort, and radiographic findings were assessed by using chi‐square analysis. The correlation between duration of cough and total radiographic score was assessed using linear regression. Significance was defined as *P* < .05.

## 3 RESULTS

Between 1 January 2011 and 1 January 2019, bronchoscopy with BAL fluid collection was performed in 99 cats. Fifty cases were excluded because of diagnoses of pneumonia (26), neoplasia (14), low BAL fluid cell count or normal cytology (8), tracheal tear (1), and upper airway obstruction secondary to severe necrotizing stomatitis (1). Forty‐nine cats were diagnosed with idiopathic IAD categorized as eosinophilic (23/49; 47%) mixed (14/49; 29%), and neutrophilic (12/24; 29%) inflammation based on BAL fluid cytology. Of the cats with mixed inflammation, 6/14 (43%) had cytologically distinct or discordant results between lavage sites, whereas the others had mixed inflammation at individual lavage sites.

**FIGURE 1:**
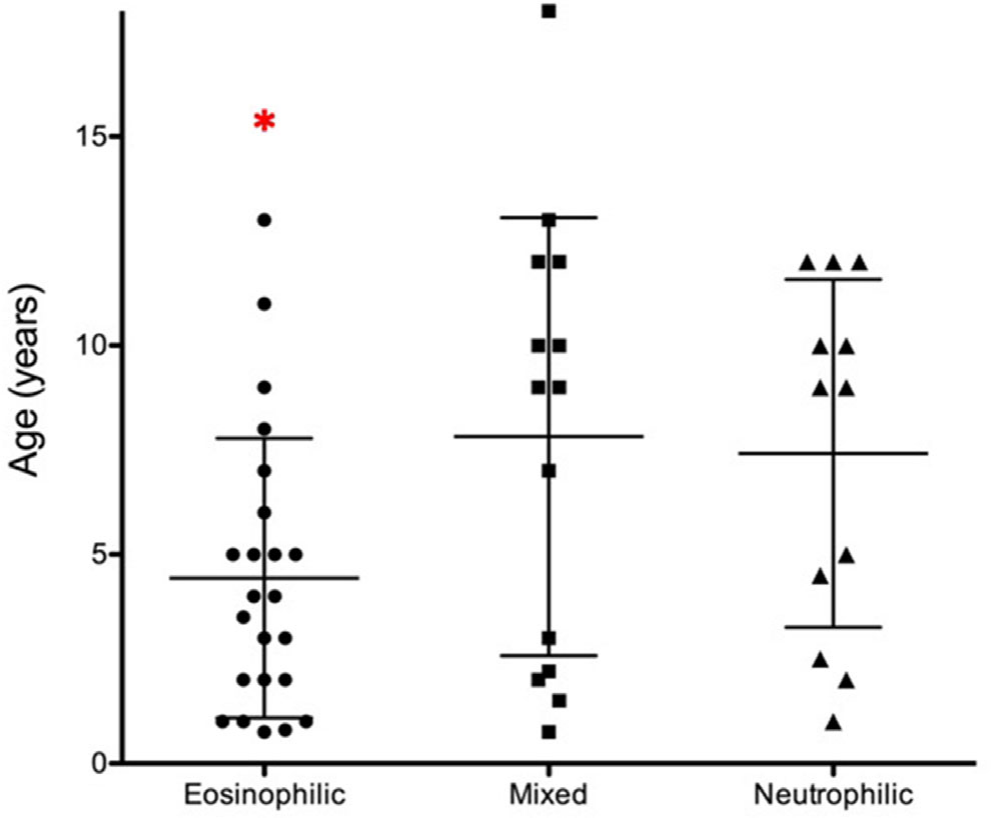
Dot plot of ages (years) of cats with eosinophilic, mixed or neutrophilic inflammatory airway disease. Means with SDs are indicated by bars. Cats with eosinophilic airway inflammation were significantly younger (*P = .03) than cats with mixed or neutrophilic inflammation

Signalment, weight, temperature, heart rate, respiratory rate, and outdoor access are presented in Table 1. Cats with IAD had a mean (±SD) age of 5.0 ± 4.4 years, weight of 4.8 ± 1.3 kg, and BCS of 5 ± 1.3. There were 28 neutered males and 21 spayed females. Breeds included domestic short hair (25), domestic medium hair (8), domestic long hair (6), Siamese (3), Devon rex (1), Abyssinian (1), Himalayan (1), Maine Coon × (1), Savannah (1), Sphinx (1), and Turkish angora (1). Cats with eosinophilic IAD were significantly younger (4.4 ± 3.3 years) than cats with mixed (8.8 ± 5.2 years) or neutrophilic inflammation (7.4 ± 4.2 years; *P* = .03; Figure 1). Indoor or outdoor status was available for 43/49 cats with 21 having access to the outdoors. No significant differences were found among groups for sex, weight, BCS, or indoor versus outdoor environment.

**TABLE 1:** Clinical information of cats with inflammatory airway disease evaluated in this study

	All (n = 49)	Eosinophilic (n = 23)	Mixed (n = 14)	Neutrophilic (n = 12)	*P* value
Age (years)	5 ± 4.3	4.4 ± 3.3*	8.8 ± 5.2	7.4 ± 4.2	.03
M/F	28/21	13/10	8/6	7/5	
Weight (kg)	4.8 ± 1.3	4.8 (3‐7.4)	4.8 (3.6‐8.7)	4.6 (3.3‐7.8)	.98
Duration of signs (months)	6 (0.03‐108)	6 (0.03‐60)	4.5 (0.1‐108)	8.5 (0.1‐48)	.76
Body temperature (°F)	101.1 (98‐106.3)	101.2 (98‐103.6)	100.9 (100.3‐106.3)	101.1 (99.2‐104.2)	.98
Heart rate	200 ± 30	192.7 ± 38.7	191.4 ± 23.4	194.0 ± 21.0	.98
Respiratory rate	54 (20‐120)	60 (20‐100)	54 (20‐80)	52 (36‐120)	.82
Outdoor access (reported in 43/49 cases)	21/43	12/21	3/12	6/10	.14

* Value is significantly different from others in the row.

The most common presenting complaint was cough 41/49 (84%) accompanied by sneezing or nasal discharge (19/49; 39%), increased respiratory effort (16/49; 33%), and wheeze (14/49; 29%), with wheeze alone in 7 cats and oculonasal discharge alone in 1 cat. Respiratory rate was recorded in 47 cats. Tachypnea (respiratory rate > 40) was noted in 34/47 (72%) cats and only 3/47 (6%) had a rate <30 breaths per minute. Sneezing or nasal discharge was reported in 11/23 (48%) cats with airway eosinophilia, 6/14 (43%) cats with mixed inflammation, and 2/12 (17%) cats with neutrophilic inflammation, with no significant differences among groups (*P* = .20). The median duration of cough for 41 cats was 5.5 months with a range of 0 days to 9 years, and no significant differences were found among groups (*P* = .76).

**FIGURE 2:**
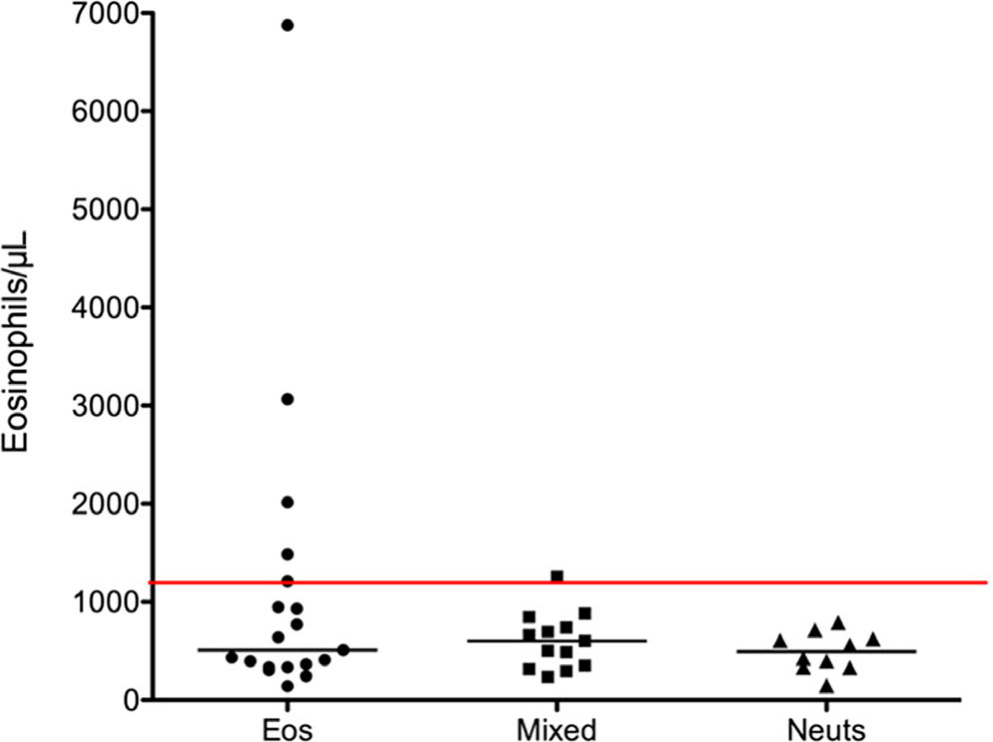
Dot plot of peripheral eosinophil counts (per μL) in cats with eosinophilic, mixed, or neutrophilic inflammatory airway disease. Medians are represented by bars, and the red line indicates the upper limit of normal peripheral eosinophil count (1100/μL)

Complete blood cell counts were available in 42 cats (Table 2). Total leukocyte counts for all cats with inflammatory disease ranged from 2937 to 28 640/μL with a median of 8425/μL. Neutrophil counts ranged from 1701 to 15 351/μL with a mean of 5434 ± 3478/μL and eosinophilic counts ranged from 140 to 6874/μL with a median of 511/μL. No significant differences were found in total leukocyte count, neutrophil count, or eosinophil count in cats with different types of BAL inflammation. Peripheral eosinophilia (>1100/μL) was present in 6 cats, 5 of which had eosinophilic disease and 1 with mixed disease, although this finding did not reach statistical significance (*P* = .38; Figure 2). Heartworm antibody testing was performed in 34/49 cats and was positive in 1 cat with eosinophilic airway inflammation, but results of antigen testing and echocardiography were both negative. Echocardiography in an additional 11 cats was negative. In comparison, heartworm testing was performed in 157 cats that did not have airway sampling performed in the hospital during the study period, and 8/157 (5%) were antibody positive and 2 were antigen positive. Three cats were positive on echocardiography and were diagnosed with clinical heartworm disease. Negative fecal flotation and centrifugation results were obtained in 5/49 cats in the study and 2 of these 5 also had negative Baermann fecal results. Fecal flotation, smear, centrifugation, or some combination of these was performed in 296 cats in the general hospital population and fecal Baermann analysis was performed in 12 cats during the study period. In these cats that did not have respiratory disorders, routine fecal flotation identified *Toxocara cati* in 3 cats and *Aelurostrongylus abstrusus* in 3 cats (1% each). Empirical fenbendazole treatment was prescribed in 16/49 cats in this study, 10/13 with eosinophilic inflammation, 3/14 with mixed, and 3/12 with neutrophilic inflammation, with no significant difference among groups (*P* = .31). Of these 16 cats prescribed fenbendazole treatment, none had a positive clinical response, and 1 patient never received fenbendazole because of lack of owner compliance. All cats subsequently responded to inhaled or PO glucocorticoids.

**TABLE 2:** Complete blood count data of 42 cats with inflammatory airway disease. Normally distributed data are presented as mean ± SD and nonparametric data are presented as median (range)

	All	Eosinophilic (n = 19)	Mixed (n = 13)	Neutrophilic (n = 10)	*P* value
White blood cells/μL	9921 (2937‐28 640)	7487 (2937‐28 640)	9160 (4030‐13 980)	9095 (4980‐16 570)	.72
Neutrophils/μL	6328 ± 3478	6125 ± 4129	5913 ± 2657	7225 ± 3212	.63
Eosinophils/μL	814 (140‐6874)	511 (140‐6874)	602 (235‐1258)	493(149‐791)	.59

**FIGURE 3:**
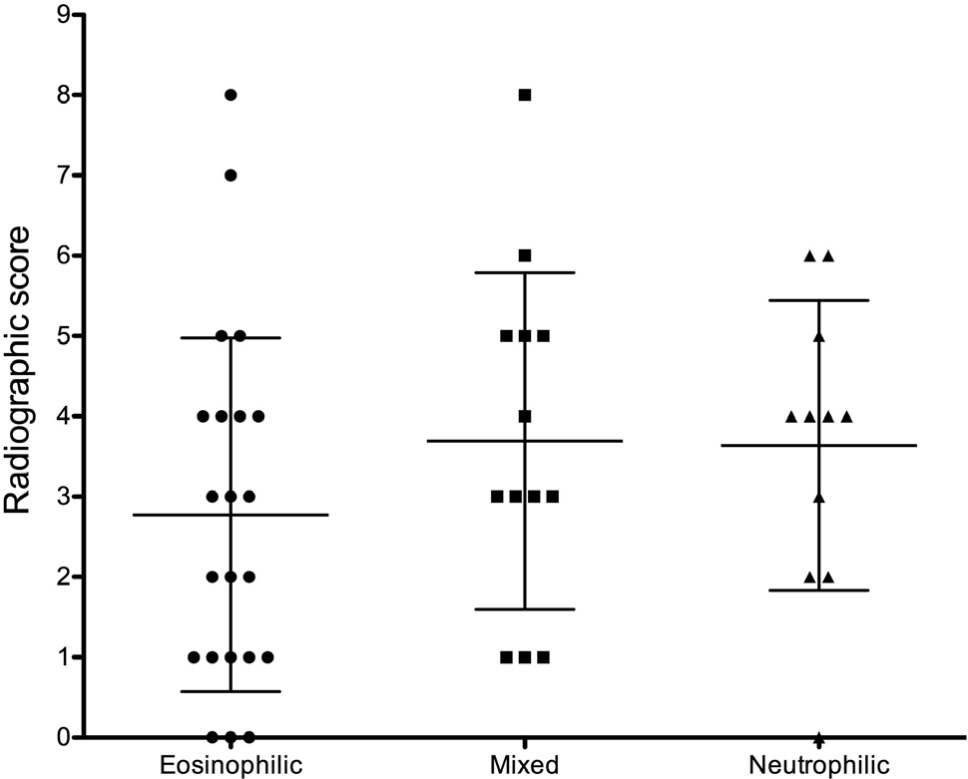
Dot plot of radiographic scores of cats with eosinophilic, mixed, or neutrophilic inflammatory airway disease. Mean and SD are indicated by bars

Thoracic radiographs were available for review in 47 of 49 cats, with most cats having multiple radiographic patterns (Table 3). Radiographs were considered normal in 3/22 cats with pulmonary eosinophilia, 1/12 cats with pulmonary neutrophilia, and none with mixed inflammation. No hyperinflation, aerophagia, or other indirect evidence of bronchoconstriction was found in these 4 cats with normal radiographs. The most common radiographic pattern was bronchial (37/47; 79%) followed by interstitial (24/47; 51%) and alveolar patterns (18/47; 38%), characterized by multifocal alveolar infiltrates consistent with mucus plugging in 10/18 and single or multiple lobar collapse in 8/18. Total radiographic score did not differ among cats with BAL fluid eosinophilic inflammation (2.8 ± 2.2), mixed inflammation (3.7 ± 2.1), and neutrophilic inflammation (3.6 ± 1.8; *P* = .30; Figure 3). Bronchiectasis was noted in 19/47 cats (40%) and broncholithiasis was visible in radiographs of 10/47 cats (21%), with no difference among groups (*P* = .13 and .64, respectively). No correlation was detected between duration of cough and radiographic score (*P* = .49).

**TABLE 3:** Radiographic findings in 47 cats with inflammatory airway disease

	All (n = 47)	Eosinophilic (n = 22)	Mixed (n = 13)	Neutrophilic (n = 12)	*P* value
Bronchial	37 (79%)	19 (86%)	11 (85%)	7 (58%)	NA
Interstitial	24 (51%)	9 (41%)	6 (46%)	8 (75%)	NA
Alveolar	18 (38%)	5 (23%)^a^	7 (54%)^b^	6 (50%)	NA
Bronchiectasis	19 (40%)	7 (32%)	7 (54%)	5 (42%)	.13
Broncholithiasis	10 (21%)	6 (27%)	2 (15%)	2 (17%)	.98

Abbreviation: NA, not applicable.

a Collapse of the right middle lobe in 2 cases, left cranial lobe in 1.

b Collapse of the right middle lobe in 3 cases, left cranial lobe in 2.

Bronchoalveolar lavage was performed at 111 sites in 49 cats, and fluid retrieval data were available in 40 cats. Mean percentage retrieval for cats with BAL eosinophilia was 73.4% ± 16.2%, 69.6% ± 21.4% for cats with airway neutrophilia, and 70.2% ± 12.2% for cats with mixed inflammation, with no difference among groups (*P* = .80). Thirty‐one cats had 2 sites lavaged, 16 had 3 sites, and 2 had 1. The most common sites of lavage were the left caudal (38/111), right caudal (36/111), and right middle (17/111) lung lobes. In 10/18 cats with alveolar patterns, lavage samples were taken from the lobe corresponding with radiographic lesions. The remaining 8 lavage samples had diffuse radiographic disease or collapsed lung lobes from which BAL samples could not be obtained because of limited scope access. No significant difference was found in the locations of sites lavaged among groups. Median TNCC did not differ among cats with different types of inflammation (*P* = .94). The percentages of inflammatory cells in the various groups of cats are presented in Table 4.

**TABLE 4:** Median with range (total nucleated cell count [TNCC]/μL) and mean with SD for cell percentages of 49 cats with inflammatory airway disease

	Eosinophilic (n = 23)	Mixed (n = 14)	Neutrophilic (n = 12)
TNCC/μL	1360 (480‐5260)	1385 (585‐11 600)	1220 (620‐9920)
% Neutrophils	16.3 ± 16.7	43.3 ± 20.8	64.8 ± 22.6
% Eosinophils	60.9 ± 16.6	35.9 ± 7.6	5.2 ± 5.7

Antimicrobials or anti‐inflammatory agents had been administered in the 7 days before airway sampling in 17 cats, with some cats receiving multiple medications. Four cats (1 with mixed, 1 with neutrophilic, and 2 with eosinophilic inflammation) had received antibiotics (a single 14 mg/kg PO dose of amoxicillin‐clavulanic acid, a single dose of amoxicillin‐clavulanic acid [12.5 mg/kg PO], amoxicillin‐clavulanic acid [14.5 mg/kg PO q12h] for 22 days and orbifloxacin [3.4 mg/kg PO q24hr] 7 days before sampling, and azithromycin [9 mg/kg PO q24hr] for the month before sampling). One cat in each inflammatory group had been treated with fluticasone propionate by inhalation. Orally administered glucocorticoids had been used in 10 cats, 4 with airway neutrophilia (1 of which also received fluticasone), 4 cats with eosinophilia, and 2 cats with mixed inflammation. Terbutaline was given to 5 cats (3 with eosinophilic and 2 with neutrophilic inflammation) and albuterol was administered to 2 cats with neutrophilic inflammation. Minimal to no response in respiratory signs was reported. Other medications administered included lysine, lantus insulin, protamine zinc insulin, and polyethylene glycol (MiraLAX, Bayer Corporation, Munich Germany) in 5 additional cats.

## 4 DISCUSSION

To our knowledge, ours is the largest study in North America evaluating clinical and radiographic features in cats with different types of naturally occurring IAD as defined by bronchoscopic BAL. A similarly large and concurrent 13‐year retrospective study from Germany utilizing both bronchoscopic and nonbronchoscopic BAL identified eosinophilic inflammation or asthma in 73 cats and neutrophilic inflammation or bronchitis in 24 cats.^13^ No differences in history, signalment, or radiographic findings were reported in that study, other than the presence of nasal discharge in cats with chronic bronchitis. In contrast, our study found that cats with BAL fluid eosinophilia were younger than cats with other types of airway inflammation, which is consistent with some^8^, ^13^, ^15^, ^20^ but not all^1^, ^6^ investigations of lower airway disease in cats. Interestingly, despite younger age, no differences in duration of signs were found among cats with different types of airway inflammation, which was contrary to our hypothesis and not specifically reported in a previous study,^13^ which required signs >3 months in duration for a diagnosis of bronchitis. Peripheral eosinophilia was relatively uncommon in our group of cats (14%) and although the highest counts tended to be in cats with respiratory eosinophilia, this result was not statistically significant. In comparison, the previous study reported peripheral eosinophilia in 40% of cats with asthma and in 27% of those with chronic bronchitis.^13^ Finally, contrary to our hypothesis, radiographic changes were of equal severity in all groups of cats and, overall, no clinical variables were found that could differentiate among cats with different types of lower airway inflammation.

Cats with eosinophilic airway disease were younger (mean age, 4.4 years) than cats with mixed or neutrophilic inflammation, which is consistent with previous reports,^8^, ^13^, ^15^, ^20^ although only 1 other study found this finding to be statistically significant.^15^ This tendency toward a younger age of onset parallels the development of asthma in humans, most commonly a childhood disease where the allergic or hypersensitivity response is related to specific development of neonatal and juvenile immunity.^21^, ^22^, ^23^ In neonatal murine models of asthma, increased diversity of lung or gut microbiota promotes a more favorable environment for tolerance against allergic disease by inducing T‐regulatory cells, lower concentrations of IgE and altered balance of Th1/Th2 immune responses.^24^, ^25^, ^26^ In studies of cats, a similar relationship has been suggested between gut and lung mucosal microbiomes.^27^, ^28^ If mucosal immunity development is the same in the cat as in the mouse, this observation could have implications for specific treatment or prevention of eosinophilic lung disease.

Airway disease in the cat also shares several corollaries with asthma syndrome in horses, which encompasses IAD, a relatively mild condition of younger horses, and recurrent airway obstruction. Both of these disorders in horses are associated with increased airway mucus and airway hyperresponsiveness, and they respond to strict control of exposure to dust and allergens. Recurrent airway obstruction is characterized by more severe airway obstruction and marked airway neutrophilia, in comparison with the mild mixed airway inflammation usually present in IAD.^29^, ^30^ Neutrophilia predominates in the allergic disease process in horses partially because of IL‐4‐mediated release of neutrophilic chemotactic factors,^31^ and the severity of disease correlates to some extent with the severity of airway neutrophilia.^32^ The cytokine milieu in the respiratory tract of cats with naturally occurring airway disease has not been evaluated, but 1 study did associate the magnitude of airway inflammation with the clinical and radiographic severity of disease,^3^ and airway responsiveness has been correlated with the severity of airway inflammation.^8^, ^15^

Airway eosinophilia was the most common IAD (47%) in our group of cats, which is similar to recent studies,^8^, ^11^, ^13^, ^15^, ^20^ but other, primarily older, publications^1^, ^2^, ^3^ have reported eosinophilic inflammation in a smaller proportion (24%‐32%) of cats evaluated. This difference might suggest an increasing trend toward eosinophilic disease in cats, which is interesting considering that allergic and asthmatic diseases have been increasing in people.^21^ This phenomenon has been attributed to multiple environmental and cultural shifts sometimes referred to as the hygiene hypothesis. It has been reported previously that exposure to rural environments has a protective effect on development of allergy and atopy in people.^33^, ^34^, ^35^, ^36^ Whether a similar situation plays a role in animals could be of interest. However, increasing recognition of eosinophilic airway disease could be a reflection of different cutoffs or definitions for eosinophilic inflammation. Older publications often defined eosinophilic airway disease based on the predominant cell type^1^, ^3^, ^19^, ^20^ with or without quantification, whereas newer publications define eosinophilic inflammation more precisely (typically >14%‐17%),^8^, ^15^, ^16^, ^17^, ^18^ with or without accounting for the severity of concurrent neutrophilic or lymphocytic inflammation.

Unsurprisingly, cough was the most common clinical complaint and was reported in 84% of cats in our study. None of the cats evaluated here were presented in respiratory distress in comparison with a recent study in which almost 1/3 of all cats had to be admitted for management of respiratory difficulty,^13^ although a majority of our cats were tachypneic with respiratory rates >40 breaths per minute. The percentage of cats with naso‐ocular discharge or sneezing (39%) was surprising. One previous study^3^ found upper respiratory tract signs in 25% of cats diagnosed with asthma or bronchitis whereas another^13^ reported nasal discharge more often in cats with chronic bronchitis than in those with asthma. An association of asthma with upper respiratory viruses has been noted in children,^37^, ^38^ and nasal airways in cats with experimentally induced asthma were noted to have eosinophilic infiltration in the absence of clinical signs.^39^ Whether this finding was related to the method of hypersensitization via aerosolized allergen in the experimental study or represented a distinct response of the upper respiratory epithelium in those cats is unclear. It seems likely that sneezing or nasal discharge noted in cats in our study was not related to the same stimulus causing lower airway inflammation.

Diffuse bronchial and interstitial patterns were the most common radiographic findings in our study, with 9% of cats having normal thoracic radiographic findings. Similar findings have been reported previously, along with the fact that radiographic findings did not differ among inflammatory categories or even between infectious and noninfectious IADs.^1^, ^2^, ^5^, ^11^, ^13^, ^15^, ^40^, ^41^ In our study, the severity of radiographic pulmonary infiltration was not associated with duration of clinical signs. Bronchiectasis was more common in this group of cats (47%) compared to previous reports of 0% to 18%.^1^, ^2^, ^5^, ^40^, ^42^ This difference could be related to the long duration of signs in all inflammatory types, but also might reflect improved detection of this finding by digital radiography. Broncholithiasis was found commonly (21%) in this group of cats. Broncholithiasis previously has been reported as a rare condition, with only 3 reports in 4 cats, all of which were presumed to have IAD.^43^, ^44^, ^45^ Lobar collapse was found slightly more often in cats examined here (17%) compared to other studies (5%‐10%).^1^, ^2^, ^5^, ^40^, ^42^ Interestingly, only cats with eosinophilic or mixed IAD were noted to have lobar collapse, which could be a result of airway narrowing or hyperresponsiveness associated with eosinophilic inflammation. As reported in other studies,^2^, ^5^, ^16^ alveolar infiltrates also were relatively common (38% of cats) in our study, although the genesis of this pattern was not established.

Contrary to our hypothesis, peripheral eosinophil counts did not differ among inflammatory cell type groups, which is consistent with previous reports.^1^, ^3^, ^5^, ^15^, ^41^ However, although the presence of peripheral eosinophilia was not statistically significant, 5 of 6 cats with peripheral eosinophilia had eosinophilic BAL inflammation and 1 had mixed inflammation. A post hoc power analysis was 48%, indicating that a larger sample size may have found significance.

Although differentiation of lower airway disease in cats into asthma or chronic bronchitis often is based on detection of eosinophilic versus neutrophilic airway lavage cytology, there is no standardized definition. In addition, multisegment BAL cytology can detect discordant inflammation in separate lung lobes of a single cat in up to 48% of cases.^16^ Given the similarities in clinical complaints, physical examination findings, clinicopathologic variables, and radiographic results among groups identified here, the previous finding of discordant BAL cytology among lobar lavage sites, and the occurrence of mixed inflammation in many cases, it is possible that asthma and chronic bronchitis in cats are different manifestations of a disease process based on inflammation. Whether this disease process has the same etiology or is multifactorial remains to be determined.

Limitations of our study are those typically encountered in a retrospective study, including lack of standardization in history collection, failure to exclude animals based on prior administration of medications, and lack of fecal or heartworm testing in all patients. Despite being in a nonendemic region for parasitic airway diseases,^46^ we cannot exclude the possibility that specific causes of airway inflammation were missed in our study. Estimation of duration of clinical signs and specific details of presenting complaints or clinical signs could have been inaccurate because of reliance on owner recollection of details. In addition, information such as specific living environment could be relevant in light of a suggested allergic etiopathogenesis with eosinophilic disease. Furthermore, not all cats had medications including corticosteroids or antibiotics withheld for >7 days before BAL, which could have resulted in less severe inflammation on BAL cytology. These cases continued to have inflammatory BAL cytology, which may indicate disease would have been more severe without treatment, although the impact of these medications on the type of inflammation detected is difficult to predict. Nevertheless, our study confirmed similarities among cats with different types of airway inflammation, with few differences discernible with the exception of younger age in cats with BAL eosinophilia. Radiographic changes tended to be severe and potentially irreversible in this group of cats, and severity did not differ with duration of signs. Further research is needed to gain additional understanding on the pathogenesis and treatment response in cats with naturally occurring IAD.

## CONFLICT OF INTEREST DECLARATION

Authors declare no conflict of interest.

## OFF‐LABEL ANTIMICROBIAL DECLARATION

Authors declare no off‐label use of antimicrobials.

## INSTITUTIONAL ANIMAL CARE AND USE COMMITTEE (IACUC) OR OTHER APPROVAL DECLARATION

Authors declare no IACUC or other approval was needed.

## HUMAN ETHICS APPROVAL DECLARATION

Authors declare human ethics approval was not needed for this study.
